# Notes and News

**Published:** 1903-03-07

**Authors:** 


### Notes and News.

### HOSPITAL MEETINGS AND FESTIVALS.

Royal Westminster Ophthalmic Hospital.—Annual Meeting, Friday, March 6, at 4 p.m.

National Hospital for Paralysed and Epileptic.—Annual General Meeting, Thursday, March 12, at 3 p.m.

There are a good many cases of small-pox still in Manchester, most of which are at the Clayton Hospital.

The Royal College of Surgeons in Ireland is issuing a medical students' guide affording all particulars of medical curriculum and specimen examination papers.

The Act making vaccination and revaccination compulsory in France will not take effect for one year. Meanwhile, some of the clauses will undergo reconsideration.

We may expect to see that the London small-pox epidemic has proved a useful object lesson to the provinces, and that the returns of persons vaccinated will probably be much larger than for some time past. This is the case at Bristol, where the vaccinations in 1902 exceeded by 1,000 those of the previous year.

Mr. Stanmore Bishop will read a paper before the British Gynæcological Society, 20 Hanover Square, on March 12th, at 8 p.m., on "Procidentea Uteri, with special reference to an Operation upon Sacro-Uterine Ligaments."

Of the 30 beds at the Royal London Ophthalmic Hospital opened by the Lord Mayor last week, 15 are in Prince of Wales ward, for men; 8 are for women, and are in Queen Victoria and other wards; and 7 are in Princess May ward, for children. The entire bedding has been provided by the Ladies' Guild connected with the hospital.

The catholicity of the London hospitals opens them sometimes to comic incidents. A gentleman of colour presented himself recently at a London hospital, but as he knew no English and spoke no language which was known at the institution, he was examined by the doctors, given a bath, had his hair cut and put carefully to bed. Next day it appeared that he was a friend of one of the patients in the hospital and had called to see how he was getting on!

Professor von Hansemann, prosector of the Friedrichshain Hospital, read a paper before the Berlin Medical Society on the subject of the conveyance of tuberculosis by food, with special reference to Dr. Koch's views concerning the non-transference of bovine tuberculosis to human beings. Professor von Hansemann's views in brief are that though tuberculosis may possibly in rare instances be conveyed by food, that pulmonary tuberculosis never results from primary intestinal tuberculosis.

The Westminster City Council is showing considerable energy in an endeavour to suppress the adulteration of food. It made recently a crusade against the use of sulphate of copper in the preservation of peas. Restaurant keepers and others know by experience that a nicely coloured pea is far more acceptable to customers who are only now being educated sufficiently in matters dietetic to inquire into the reason why some peas are of a tempting green, whilst others which taste equally well appear most unattractive. As preserved peas have always been used for decorative purposes in a menu, they will soon go out of fashion unless a harmless colouring process can be discovered.

We are requested by the Secretary of the League of Mercy to announce that Sir Henry Burdett, K.C.B., will deliver an address on "Life in the Hospitals" on behalf of the League, at the Borough Hall, Greenwich, on Friday, March 13th, at 8 p.m., and at the Large Hall, Kensington Town Hall, on Thursday, March 19th, at 8.30 p.m. Sir William Collins, M.D., one of the hon. secretaries, will speak at a meeting to be held in the Parish Room, Enfield, on Friday, March 6th, to further the objects of the League of Mercy, which are to promote the welfare of King Edward's Hospital Fund for London in every way, but especially by encouraging personal service, and to advance the interests and contribute towards the adequate maintenance of hospitals.

